# Internal consistency and correlation of the adverse childhood experiences, bully victimization, self-esteem, resilience, and social support scales in Nigerian children

**DOI:** 10.1186/s13104-020-05174-3

**Published:** 2020-07-10

**Authors:** Morenike Oluwatoyin Folayan, Olakunle Oginni, Olaniyi Arowolo, Maha El Tantawi

**Affiliations:** 1 Obafemi Awolowo University Teaching Hospitals Complex, Ile-Ife, Nigeria; 2 Department of Child Dental Health, Obafemi Awolowo University, Ile-Ife, Nigeria; 3 Faculty of Dentistry, Alexandria University, Alexandria, Egypt

**Keywords:** Mental health, Internal consistency, Adverse childhood experiences, Bully victimization, Self-esteem, Resilience, Social support, Psychometric scales, Children, Adolescents, Nigeria

## Abstract

**Objectives:**

We evaluated the internal consistencies and the correlation between measures of adverse childhood experiences (ACE), bully victimization, self-esteem, resilience, and social support in children/adolescents in Nigeria.

**Results:**

The Cronbach’s alphas were 0.67 for the ACE Questionnaire; 0.79 for the victimization subscale of the Illinois Bully Scale; 0.60 for Rosenberg’s self-esteem scale; 0.81 for Connor–Davidson resilience scale; and 0.93 for multidimensional perceived social support scale. Social support was negatively correlated with ACE (*r* = − 0.21) and bully victimization (*r* = − 0.16) and was associated with higher self-esteem (*r* = − 0.29) and higher resilience (*r* = 0.15). Likewise, higher resilience was associated with fewer ACE (*r* = − 0.07), higher self-esteem (*r* = − 0.21), and higher bully victimization (*r* = 0.13). Higher self-esteem was associated with fewer ACE (*r* = 0.25) and lower bully victimization (*r* = 0.16), whereas bully victimization was positively correlated with ACE (*r* = 0.20). The correlations were all statistically significant.

## Introduction

Poor mental health is a major health problem affecting children and adolescents globally. Mental health disorders have great impact on children ages 5 to 14 years in North America and Europe, and they are a growing problem in other regions of the world due to the complex change in patterns of health and disease and the interaction with demographic, economic, and sociologic determinants [1].

Factors that affect children’s and adolescents’ mental health include adverse childhood experiences (ACE) and bully victimization. ACE include physical or sexual abuse; domestic violence; exposure to violence in the community; neglect; the absence or limited availability of a caregiver; poverty; and food insecurity [2]. These experiences affect emotional expression and increase the use of maladaptive emotion-regulation strategies [3, 4]. These coping measures can result in psychopathology such as depression, anxiety, aggressive behavior, post-traumatic stress disorder, and substance abuse [5–7]. Children and adolescents who experience bully victimization may face these mental health challenges also in adult life [8–11].

Factors that protect against mental health problems include self-esteem, resilience, and social support. Self-esteem is the ability to hold a favorable attitude towards one’s self. High self-esteem is associated with fewer symptoms of anxiety and depression [12]. Resilience, which is the ability to positively adapt to adversity, is associated with good mental health [13–15]. Also, previous studies showed that the support that individuals perceive they get through social interactions moderates the impact of ACE and bully victimization through a buffering effect that reduces psychological distress when stressful situations develop [12, 16, 17].

Though the impact of ACE, bully victimization, self-esteem, resilience and social support on mental health is assumed to be a global phenomenon [18], the interactions between these variables may vary with culture (beliefs, norms and values) [19]. Most of the studies on ACE, bully victimization, self-esteem, resilience, social support, and mental health have been conducted in Western countries, which are generally individualistic societies and differ in many ways from the collectivist societies of Africa [20].

ACE, bully victimization, and adolescents’ concern with self-esteem are also problems in Nigeria [21–24], and mental health difficulties are a growing problem among adolescents in the country [25]. It is important to conduct more studies, using validated tools, to elucidate the correlations between variables associated with mental health problems in settings and adolescent populations in Nigeria. The aim of this study was therefore to assess the internal consistency of tools used to measure ACE, bully victimization, self-esteem, resilience, and social support in a population of in-school children and adolescents in sub-urban Nigeria. We also determined how the constructs correlated with one another. We hypothesized that ACE and bully victimization would be associated negatively with social support, resilience, and self-esteem.

## Main texts

Data were collected as part of a cross-sectional study that determined the association between caries and nutritional status in 6–16-year-old children attending private and public primary and secondary schools in Ife Central Local Government Area, Ile-Ife, Osun State, Nigeria. Data were collected from October to December 2019. Children at least 6-year old were recruited to ensure they had the cognitive ability to respond to the interviewer-administered questionnaire [26, 27]. Recruiting children 6–10-year-old for study participation further reduces the risk for recall bias, a major challenge when studying the impact of ACE and bullying in older individuals [28]. Children and adolescents with special health-care needs, those who were ill, and those who had fasted within 3 months before data collection, were excluded from the study.

### Sample size and sampling procedure

The sample size for the primary study was 1209. Post-hoc power analyses using the formula by Metcalfe [29] indicated a power of 80% given a population prevalence rate of 13.9% for caries in children [30]. The sample was collected through a multi-stage cluster sampling technique that recruited 6–10-year-old children from primary schools, and 11–16-year-old children from secondary schools. Twenty primary schools (3 public, 17 private) and 10 secondary schools (2 public, 8 private) were randomly selected from the list of schools in the local government based on the ratio of public/private primary and public/private secondary schools. Data were collected from pupils using tools written in English and administered by interviewers; English is the language of instruction in schools in Nigeria.

*Adverse childhood experiences* were measured with the 10-item ACE Questionnaire, which provides a measure of cumulative life stress experienced during childhood [31]. The response to each of the 10 questions is either ‘yes’ or ‘no,’ with possible scores of 0 to 10. The higher the score the more adverse experiences the child has faced. Although the instrument had not been validated for use in Nigeria, validation in other countries indicated that it had good psychometric properties, with high correlation between mental and physical health measures and childhood trauma inventories [32].

*Childhood bully victimization* was assessed with the victimization subscale of the Illinois Bully Scale [33]. The subscale consists of four questions that measure both physical and verbal victimization that individuals experience from or by peers. The responses to each question ranged from never (scored 0) to 1–2 times (1), 3–4 times (2), 5–6 times (3), and 7 or more times (4). The responses were summed to derive a total score that ranged from 0 to 16. The verbal bullying, physical bullying, and bully victimization subscales of the Illinois Bully Scale were validated among 394 pupils in two secondary school in semi-urban southeast Nigeria. The Cronbach’s alphas for the whole scale and the victimization subscale were 0.84 and 0.78, respectively [17].

*Self*-*esteem* was assessed with the 10-item Rosenberg’s self-esteem scale that evaluated whether respondents’ perceptions of themselves were positive or negative. Items were scored on a Likert-like scale with options ranging from “Strongly Disagree” (1 point), “Disagree” (2 points), “Agree” (3 points) to “Strongly Agree” (4 points). The total score ranged from 10 to 40. Five item scores were reversed so that higher scores of all items in the scale indicated lower self-esteem. The scale had good psychometric properties [34] and was validated among 300 male and female senior secondary adolescents randomly selected from 15 secondary schools in Ile-Ife, Nigeria. The test–retest reliability score ranged from 0.85 to 0.88 [35], but no Cronbach’s alpha score had been previously reported among Nigerian children and adolescents.

*Resilience* was assessed with the 10-item Connor–Davidson resilience scale with each item rated on a 5-point scale from 0 (‘not true at all’) to 4 (‘true nearly all the time’). Possible total scores range from 0 to 40 with higher scores indicating higher resilience [36]. The scale had good psychometric properties for use in various cultures [37, 38]. It was validated with nursing students in Nigeria, with a Cronbach’s alpha of 0.81 [39], but it has not been previously used among Nigerian children and adolescents.

*Social support* was assessed with the 12-item multi-dimensional perceived social support scale [40]. The scale has three subscales that assess an individual’s perception of the adequacy of their support from family, friends, and significant others. Each subscale comprises four questions. Each item is rated on a 7-point Likert-like scale ranging from 1—“very strongly agree” to 7—“very strongly disagree.” The possible total scores range from 12 to 84, with higher total scores corresponding to higher levels of perceived social support and lower scores corresponding to perceived unavailability or lack of social support. The scale had a Cronbach’s alpha of 0.80 when administered to secondary school students in semi-urban Southeast Nigeria [17]; a Cronbach’s alpha of 0.78 when administered to stroke survivors attending rehabilitation services in Kano, Northern Nigeria [41]; and a Cronbach’s alpha of 0.67 when administered to Orthopaedic patients in Enugu, Southeast Nigeria [42].

### Data analysis

The internal consistency was determined using Cronbach’s alpha for the 10-item ACE Questionnaire, victimization subscale of the Illinois Bully Scale, 10-item Rosenberg’s self-esteem scale, 10-item Connor–Davidson resilience scale, and the 12-item multidimensional perceived social support scale. Pearson’s correlation coefficient (*r*) was calculated to determine correlations among the scales. Statistical analyses were conducted with Stata/SE 14.0 for Windows (2015) and significance was set at *p *< 0.05.

## Results

The study population consisted of 1001 pupils, of which 549 (54.8%) were girls and 962 (96.1%) were 11–16 years old. The mean (standard deviation-SD) age was 13.4 (1.77) years. Cronbach’s alphas were 0.67 (95% CI 0.64, 0.70) for the ACE Questionnaire; 0.79 (95% CI 0.77, 0.81) for the victimization subscale of the Illinois Bully Scale; 0.60 (95% CI 0.56, 0.64) for the 10-item Rosenberg’s self-esteem scale; 0.81 (95% CI 0.79, 0.83) for the 10-item Connor–Davidson resilience scale; and 0.93 (95% CI 0.92, 0.94) for the 12-item multi-dimensional perceived social support scale.

Table 1 shows the correlation between the psychosocial factors. All the factors had low but statistically significant correlations with one another. The directions of these correlations differed, however: The direction of the correlation between social support and ACE and bully victimization were inverse; the higher the perceived social support, the lower the number of ACE (*r *= − 0.21), and the lower the bully victimization (*r* = − 0.16). In contrast, the higher the social support, the higher the self-esteem (*r *= − 0.29) and the higher the resilience (*r *= 0.15).

**Table 1:** Correlation between the psychosocial variables

Psychosocial factors	ACE	Bully victimization	Self-esteem	Resilience
Bully victimization	0.20***			
Self esteem	0.25***	0.16***		
Resilience	− 0.07*	0.13***	− 0.21***	
Social support	− 0.21***	− 0.16***	− 0.29***	0.15***

*p < 0.05, ***p < 0.001

Higher resilience was significantly associated with fewer ACE (*r* = − 0.07), higher self-esteem (*r *= − 0.21) and higher bully victimization (*r *= 0.13). Self-esteem were significantly and directly correlated with the number of ACE and with bully victimization: self-esteem was lower among participants with more ACE (*r *= 0.25) and those with higher bully victimization (*r* = 0.16). Similarly, bully victimization significantly increased as the number of ACE increased (*r *= 0.20).

Cronbach’s alphas for the victimization subscale of the Illinois Bully Scale, the 10-item Connor–Davidson resilience scale, and the 12-item multidimensional perceived social support scale were high and compared well with values previously obtained in Nigeria, despite being administered to a more diverse population than our current study population [39, 41, 42]. This similarity suggests that the internal consistency of these instruments is stable across various populations. The Cronbach’s alpha values for the ACE questionnaire and the 10-item Rosenberg’s self-esteem scale were moderate. These estimates from the present study are the first Cronbach’s alpha values for these instruments to be reported among children and adolescents in Nigeria.

There were significant relationships between the study variables as predicted by our hypothesis, though bully victimization correlated positively with resilience. Though it has been suggested that low levels of stress can trigger adaptive coping strategies, which may increase resilience [43], the study finding of a positive correlation between bully victimization and resilience needs to be specifically tested.

In contrast, the negative association between resilience and ACE suggests different mechanisms of protection conferred by resilience for different childhood experiences. Considering the positive associations between resilience, self-esteem and social support found in the present study, it is also possible that resilience and social support together minimize the adverse impacts of stresses in children and adolescents. However, these protective effects may be stronger for bully victimization while the adverse impacts of ACE on children and adolescents’ resilience may be relatively more severe. For example, children may feel less threatened by bullying from their peers compared to maltreatment from older adults, over which they may have less control, but this possibility needs to be explored. The association between low social support and increased bully victimization suggests that the isolation associated with lower social support [44] may make affected children targets for bully victimization. It is important, however, to interpret these findings cautiously as this was a cross-sectional study.

Self-esteem score directly correlated with ACE and bully victimization indicating a negative relationship in view of the reverse scoring system for self-esteem: children exposed to more ACE and bully victimization had lower self-esteem than did others not exposed or exposed less to ACE and bully victimization. This finding corroborates past observations [45] and raises concern about the mental health problems of those exposed to ACE and bully victimization. Possible mental health problems resulting from low self-esteem include problems with friendships and romantic relationships, impaired academic and job performance, and increased vulnerability to drug and alcohol abuse [46]. Harnessing the potential of the social support systems in these communities to ameliorate the impact of ACE and bully victimization is as important as instituting efforts to reduce children’s experiences of these events.

## Limitations

The ACE Questionnaire and the 10-item Rosenberg’s self-esteem scale had moderate internal consistency, so they may require modification or cultural adaptation to improve their Cronbach’s alpha scores. In addition, though the Cronbach’s alpha provides an estimate of internal consistency, it does not provide an exhaustive indication of a measure’s psychometric properties [47]. However, considering that the assessed constructs are relevant for health research and policy development, our findings offer preliminary evidence of acceptable reliability, which can be investigated in future studies. Furthermore, the instruments need to be translated into the main languages used in Nigeria to make them more useable among non-literate Nigerians.

## Data Availability

The data for the study have all been reported in the manuscript.

## Abbreviation

ACE: Adverse childhood experiences
